# Efficient Antibacterial Membrane based on Two-Dimensional Ti_3_C_2_T_x_ (MXene) Nanosheets

**DOI:** 10.1038/s41598-017-01714-3

**Published:** 2017-05-09

**Authors:** Kashif Rasool, Khaled A. Mahmoud, Daniel J. Johnson, Mohamed Helal, Golibjon R. Berdiyorov, Yury Gogotsi

**Affiliations:** 1 Qatar Environment and Energy Research Institute (QEERI), Hamad Bin Khalifa University (HBKU), P.O. Box 5825, Doha, Qatar; 2 Department of Materials Science and Engineering and A.J. Drexel Nanomaterials Institute, Drexel University, Philadelphia, PA 19104 USA

## Abstract

Advanced membranes that enable ultrafast water flux while demonstrating anti-biofouling characteristics can facilitate sustainable water/wastewater treatment processes. MXenes, two-dimensional (2D) metal carbides and nitrides, have attracted attention for applications in water/wastewater treatment. In this work, we reported the antibacterial properties of micrometer-thick titanium carbide (Ti_3_C_2_T_x_) MXene membranes prepared by filtration on a polyvinylidene fluoride (PVDF) support. The bactericidal properties of Ti_3_C_2_T_x_ modified membranes were tested against *Escherichia coli* (*E*. *coli*) and *Bacillus subtilis* (*B*. *subtilis*) by bacterial growth on the membrane surface and its exposure to bacterial suspensions. The antibacterial rate of fresh Ti_3_C_2_T_x_ MXene membranes reaches more than 73% against *B*. *subtilis* and 67% against *E*. *coli* as compared with that of control PVDF, while aged Ti_3_C_2_T_x_ membrane showed over 99% growth inhibition of both bacteria under same conditions. Flow cytometry showed about 70% population of dead and compromised cells after 24 h of exposure of both bacterial strains. The damage of the cell surfaces was also revealed by scanning electron microscopy (SEM) and atomic force microscopy (AFM) analysis, respectively. The demonstrated antibacterial activity of MXene coated membranes against common waterborne bacteria, promotes their potential application as anti-biofouling membrane in water and wastewater treatment processes.

## Introduction

Bactericidal nanomaterials are widely explored effectively in public health applications including medical devices, water treatment, food packaging, and in the textile industries^1–3^. Among a wide spectrum of nanomaterials with proven bactericidal efficacy, antibacterial properties of 2D nanosheets, including MXenes, graphenes and MoS_2_ have been explored to meet these challenges^4–6^. Several groups have also shown that decorating nanoparticles on the surface of 2D architectures including graphene oxide (GO) increases its antimicrobial effect^7–9^. The antimicrobial activities of MoS_2_ and graphene-based materials, including graphite, graphite oxide, graphene oxide (GO), and reduced GO (rGO), against Gram-negative and Gram-positive bacteria have been found to be the synergy of both “chemical” and “physical” factors^6, 10–16^. Most of the above studies have attributed the antibacterial activity of GO and rGO to cellular membrane stress induced by sharp edges of graphene nanosheets, which may result in physical damage of cell membranes, leading to a loss of bacterial membrane integrity^2, 17–20^.

MXenes are a family of two-dimensional (2D) transition metal carbides and nitrides with a common formula of M_n+1_X_n_T_x_, where M is an early transition metal, X is C and/or N, n = 1, 2 or 3, and T_x_ represents surface functional groups, such as F, OH, or O^21^. MXenes combine a hydrophilic surface, metallic conductivity, and a high capacity for ion adsorption, which was proven by the reversible intercalation of cations (e.g., Li^+^, Na^+^, K^+^, Mg^2+^, etc.). These properties render MXene a promising candidate for environmental remediation applications^21^. Ti_3_C_2_T_x_ MXene has been widely explored in several applications including heavy metal adsorption^22–24^ and photodegradation of dyes^25^. A biocompatible composite based on soybean phospholipid modified Ti_3_C_2_ nanosheets was recently used for cancer therapy^26^. Highly flexible and ionically conductive MXene membranes with layered nanosheets showed selective sieving of high valence ions^27^. Recently, Ti_3_C_2_T_x_ membranes with controlled thicknesses, flexibility, and high mechanical strength with unique separation properties were revealed^28^. According to Ding *et al*., a MXene membrane with stacked thin layers achieved a high rejection rate (90%) for large size molecules (>2.5 nm) while maintaining over 1000 L m^2^ h^−1^ bar^−1^water permeance^29^. A major success indicator for any water treatment membrane is the resistance to biofouling caused by living organisms. Colloidal Ti_3_C_2_T_x_ showed high antibacterial properties against *E*. *coli* and *B*. *subtilis*, as confirmed by scanning electron microscopy (SEM) and transmission electron microscopy (TEM) coupled with lactate dehydrogenase (LDH) release assay indicated the damage to the bacterium cellular membrane^5^. Nevertheless, biofouling is a major limitation for separation membrane development^30^. Bacteria and other microorganisms adhere to the membrane surface and form a viscous gel-like biofilm causing a severe decline in flux^30^.

Here, in an effort to advocate the potential of 2D metal carbides for use in water purification membranes, we investigate for the first time the antibacterial activity of Ti_3_C_2_T_x_ modified membranes by taking into consideration the bactericidal activity of the colloidal Ti_3_C_2_T_x_ shown in our earlier study^5^. The antimicrobial activities of various membranes were investigated against Gram-negative and Gram-positive bacteria by filtering certain concentrations of bacterial suspensions through the PVDF and PVDF-supported Ti_3_C_2_T_x_ membranes. The latter were fabricated by vacuum-assisted filtration (VAF) and their ability to inhibit *E*. *coli* and *B*. *subtilis* bacterial growth was studied. The interactions between MXene 2D nanosheets and bacteria have been investigated by SEM, AFM, and flow cytometery.

## Results and Discussions

### Physical and chemical characterization of Ti_3_C_2_T_x_ based membranes

**Figure 1:**
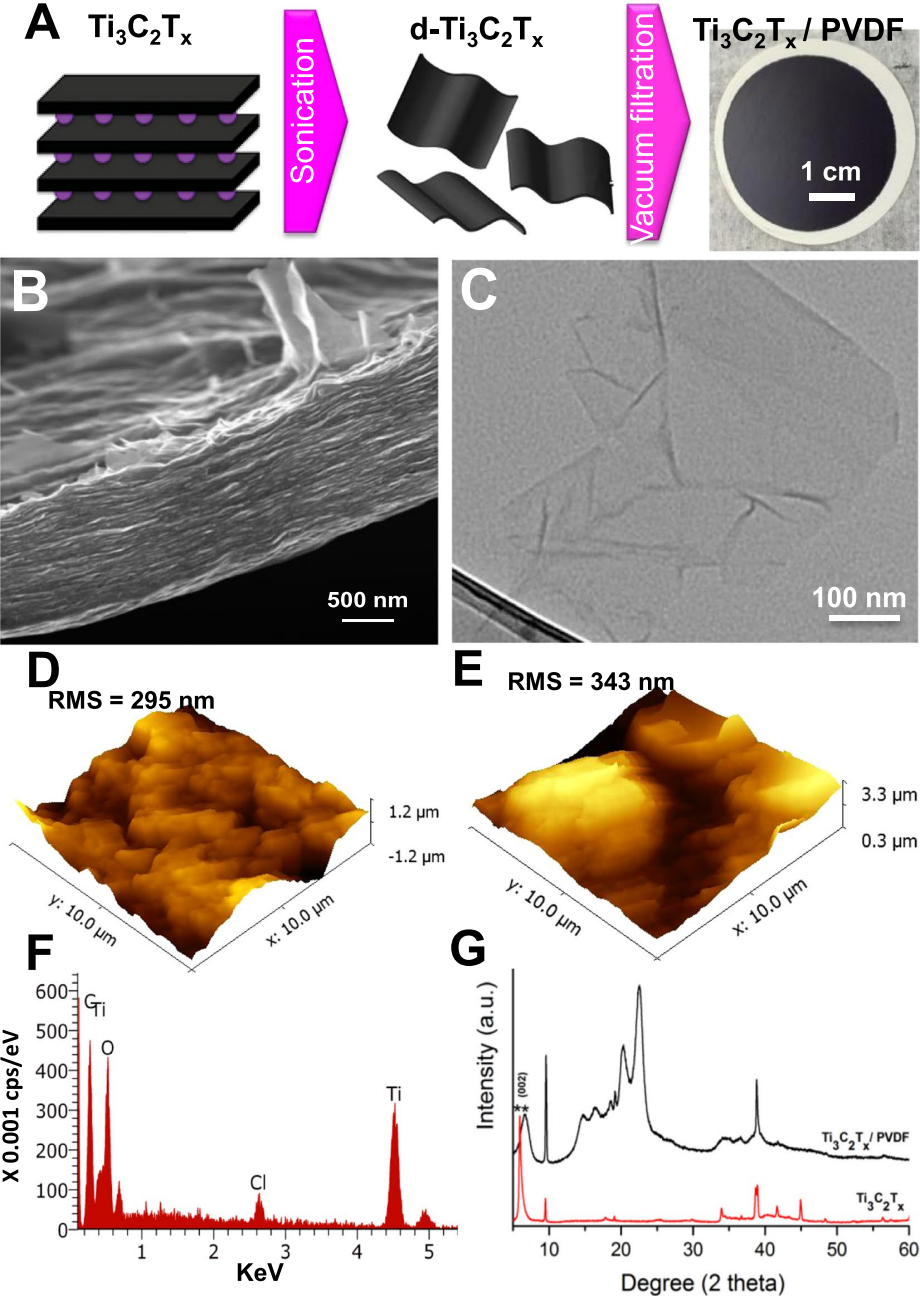
(**A**) Schematic of the Ti_3_C_2_T_x_ membrane fabrication on a PVDF support; (**B**) Cross-sectional SEM image of a Ti_3_C_2_T_x_ film; (**C**) TEM image of a pristine Ti_3_C_2_T_x_ flake; (**D** and **E**) 3D AFM images of a PVDF membrane and a Ti_3_C_2_T_x_/PVDF film, respectively; (**F**) EDX spectrum of Ti_3_C_2_T_x_/PVDF; (**G**) XRD patterns of Ti_3_C_2_T_x_ and a Ti_3_C_2_T_x_/PVDF membrane.

In order to obtain uniform film coating on PVDF as shown in Fig. 1A, a dilute colloidal Ti_3_C_2_T_x_ solution (~0.01 mg/mL) was used^31^. The dilute solutions contained primarily single-layer Ti_3_C_2_T_x_ sheets with thickness on the order of 1 nm and lateral sizes on the order of hundreds of nanometers to several microns^32^. The high aspect ratio of the nanosheets ensures uniform and narrow 2D nanochannels and mitigates the presence of meso- and macro-pores across the membrane (Fig. 1B). TEM image in Fig. 1C is showing a single flake of delaminated Ti_3_C_2_T_x_, with lateral sizes up to a few hundred nanometers. Considering the relatively high pressure exerted on Ti_3_C_2_T_x_ membranes during the experimental procedure, commercial polyvinylidene fluoride (PVDF) supports (450 nm pores) were used^31^. The hydrophilicity of the membrane was evaluated by measuring the water contact angle of pristine PVDF membranes and after coating with Ti_3_C_2_T_x_ film. PVDF was hydrophobic with a contact angle of 81°. On the other hand, Ti_3_C_2_T_x_ coated membrane functionalization significantly increased the hydrophilicity of the membrane, decreasing the water contact angle to 37°. AFM was used to compare the surface roughness patterns of pristine PVDF and Ti_3_C_2_T_x_/PVDF membranes (Fig. 1D,E). Upon Ti_3_C_2_T_x_ coating, the membrane surface became rougher with RMS value increasing from 295 to 343, due to MXene wrinkles and edges that are also seen in the SEM image (Fig. 1B). Energy-dispersive X-ray spectroscopy (EDS) analysis confirmed the presence of Ti_3_C_2_T_x_ in the elemental composition onto the PVDF surface indicated by signature Ti peak at 4.5 KeV (Fig. 1F). X-ray diffraction (XRD) patterns of air-dried Ti_3_C_2_T_x_ powder and Ti_3_C_2_T_x_/PVDF membrane are shown in Fig. 1G. The large (00 *l*) peaks at 5.8 degree from basal planes of MXene are characteristic of delaminated Ti_3_C_2_T_x_ with layers of water between the sheets. The peak of Ti_3_C_2_T_x_/PVDF is broader than pristine delaminated Ti_3_C_2_T_x_ and has been shifted to a higher 2θ angle, from 5.8 to 6.6 degree, corresponding to a decrease of the *c*-lattice parameter for Ti_3_C_2_T_x_ on PVDF as compared with pristine Ti_3_C_2_T_x_^33, 34^, indicating a decrease of interlayer spacing in the MXene film on PVDF^28^. Characteristic peaks of Ti_3_C_2_T_x_ from 18 to 40° are still observed, which suggests a good periodicity between the stacked MXene layers. Other prominent peaks from the Ti_3_C_2_T_x_/PVDF membrane are characteristic of the supporting PVDF^35^.

### Antibacterial activity of Ti_3_C_2_T_x_ membrane

**Figure 2:**
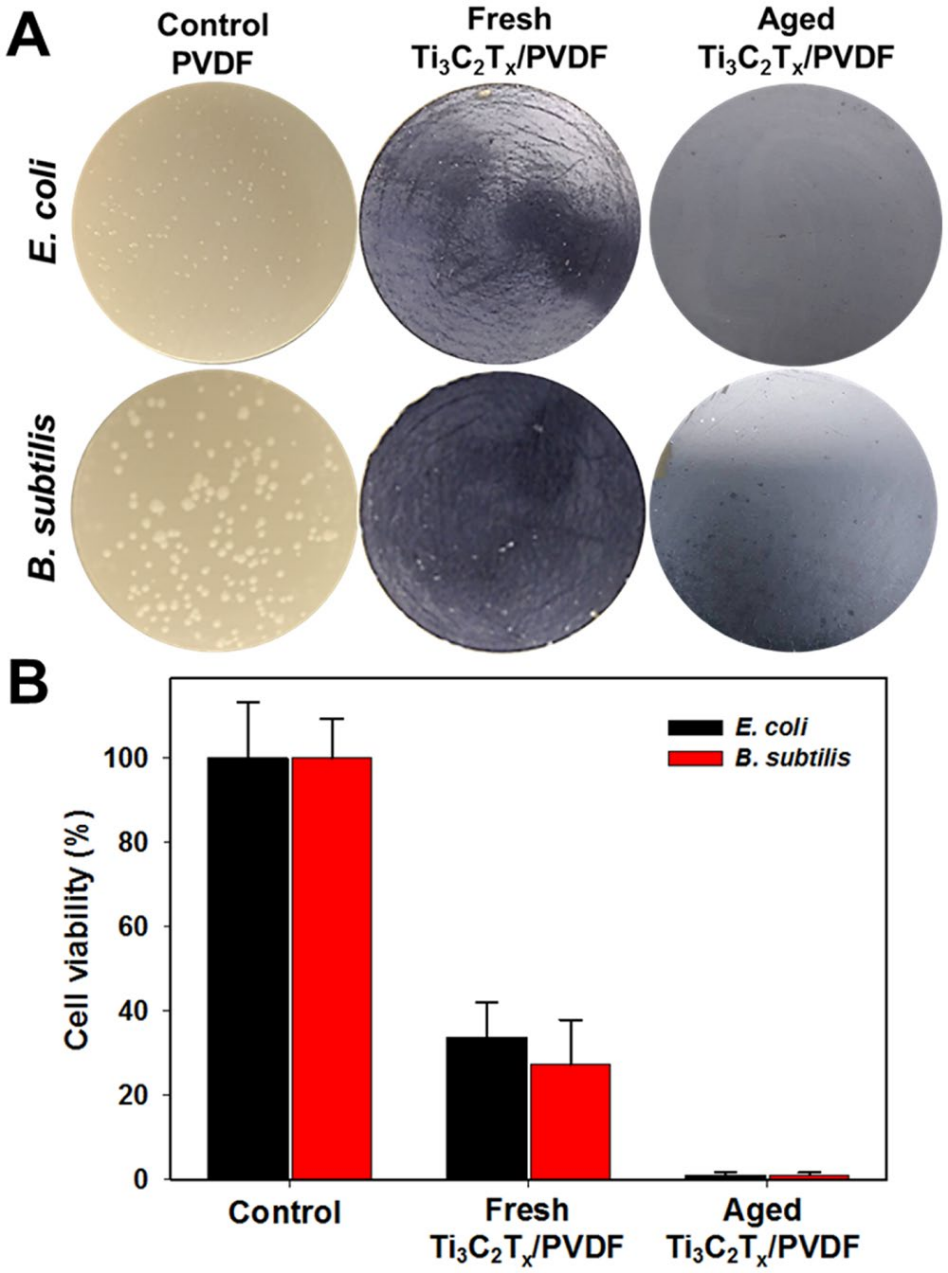
Antibacterial activity of Ti_3_C_2_T_x_ MXene. (**A**) Photographs of *E*. *coli* and *B*. *subtilis* growth on unmodified PVDF (control), and fresh and aged Ti_3_C_2_T_x_ MXene coated PVDF membranes incubated at 35 °C for 24 h. (**B**) Cell viability measurements of *E*. *coli* and *B*. *subtilis* grown on fresh and aged Ti_3_C_2_T_x_ MXene coated PVDF membranes for 24 h. Survival rates were obtained by the colony forming count method. Error bars represent the standard deviation of triplicate experiments.

Figure 2 depicts the antibacterial activities of Ti_3_C_2_T_x_ coated membranes against *E*. *coli* and *B*. *subtilis* as compared with the PVDF control after being used for filtering 10^4^ CFU.mL^−1^ bacterial solution. A significant decrease in count for viable colonies of both bacteria on membrane surface after 24 h incubation with Ti_3_C_2_T_x_ modified membranes was observed (Fig. 2A). Therefore, Ti_3_C_2_T_x_ films were able to inhibit bacteria growth and reduce viability of bacterial cells as compared with pristine PVDF membranes. The growth inhibition of Ti_3_C_2_T_x_ membranes reaches about 73% against *B*. *subtilis* and 67% against *E*. *coli* as compared to that of control PVDF (Fig. 2B). This shows the antibacterial activity of Ti_3_C_2_T_x_ coated membranes against both Gram-positive and Gram-negative bacterial strains. Differences in antibacterial activity against *E*. *coli* and *B*. *subtilis* can be related to their different cell wall structure. For instance, Gram-negative *E*. *coli* have a thin layer of peptidoglycan (2–3 nm) between the inner and outer cell membranes, whereas Gram-positive *B*. *subtilis* have a thicker peptidoglycan layer (20–80 nm), resulting in a higher resistance towards Ti_3_C_2_T_x_ film. Additionally, there was no growth of bacteria in the infiltrate through both PVDF and Ti_3_C_2_T_x_/PVDF membranes assuming 100% retention. Another set of experiments was performed to investigate the effect of Ti_3_C_2_T_x_ film thickness on its antibacterial activity. As expected, increasing the Ti_3_C_2_T_x_ thickness from 0.6 to 1.8 µm showed no significant effect on antibacterial activity (Supplemental Material, Figure. S1), indicating that the bacteria are exposed to the surface layer of the membrane. 1.2 µm Ti_3_C_2_T_x_ coating has been used in this study as an optimal membrane thickness. In our earlier study, 1.2 µm Ti_3_C_2_T_x_ coating demonstrated ultrafast water flux of 37.4 L · Bar^−1^ · h^−1^ · m^−2^ with charge selective ions sieving depending on both the hydration radius and charge of the ions^28^. Antibacterial activity of Ti_3_C_2_T_x_ nanosheets in suspension has been elaborated in our earlier study^5^, however, this is the first report to evaluate the antibacterial activity of Ti_3_C_2_T_x_ MXene coated membranes.

Effect of environmental condition on membrane efficiency is also an important factor. So it was initially assumed that oxidation of the membrane surface and formation of titanium oxide may decrease the antibacterial activity of the membrane surface. Controlled oxidation of Ti_3_C_2_T_x_ in air results in the formation of anatase TiO_2_ nanocrystals embedded in amorphous TiO_2_-C^36–39^. Ghassemi *et al*., reported the formation of thin anatase nanoparticles, and the sheets of nanocrystalline rutile by oxidation of top and bottom Ti layers under the flash and slow oxidation regimes, respectively^40^. XPS studies revealed that aged MXene films were surrounded by a thin layer of oxides together with graphitic carbon, which helped to maintain conductive contact between MXene particles^41^. The amount of oxide is dependent on the exposure time to ambient air^32^. To investigate the impact of membrane stability on the bactericidal efficiency, fresh membranes were stored at ambient air and room temperature for over 30 days. After which, the antibacterial activity of aged Ti_3_C_2_T_x_ membranes was investigated in the same manner described above. As shown in Fig. 2, the growth of bacterial colonies on the aged Ti_3_C_2_T_x_ membranes was hindered as compared to fresh membranes: >99% bacterial growth inhibition of both *E*. *coli* and *B*. *subtilis* was observed for aged membranes as compared to fresh membranes which showed 73% growth inhibition for *B*. *subtilis* and 67% for *E*. *coli*. This is a very important finding showing that aging of the membrane is advantageous to enhance the overall antibacterial properties. This could be attributed to the presence of TiO_2_ nanocrystals on the Ti_3_C_2_T_x_ membrane surface. Similarly, titanium substrates coated with nanostructured TiO_2_ showed a significant reduction in *E*. *coli* accumulation over large areas^42^.

**Figure 3:**
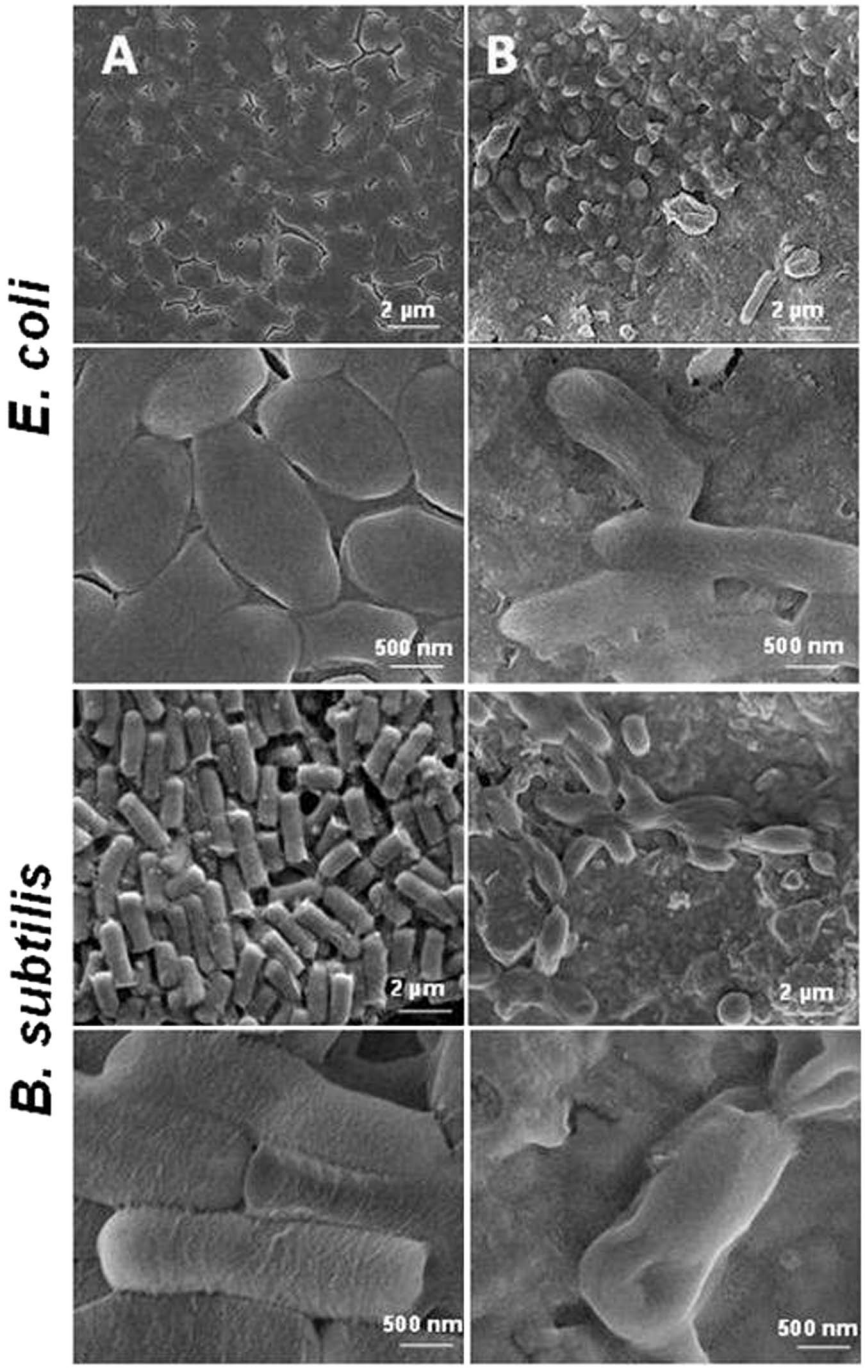
SEM images of the *E*. *coli* (top panels) and *B*. *subtilis* (bottom panels) colonies grown on (**A**) PVDF and (**B**) Ti_3_C_2_T_x_ modified membranes, at low and high magnifications. Control bacterial cells were viable with no observed membrane damage or cell death, and the higher magnification images show that the bacteria were protected by intact cytoplasmic membrane.

To further investigate the interaction of the membrane surface with bacteria, the surface morphologies of uncoated PVDF and Ti_3_C_2_T_x_/PVDF membranes after 24 h of incubation were examined by SEM (Fig. 3). The bacterial cells on Ti_3_C_2_T_x_ modified membranes showed evident differences compared to the control PVDF. Cell density of *E*. *coli* and *B*. *subtilis* grown on Ti_3_C_2_T_x_ modified membranes was significantly lower as compared to that of control PVDF membranes. It was also found that the cells on the pristine PVDF surface were smooth, intact and viable without any membrane disruption. However, few bacterial cells survived on Ti_3_C_2_T_x_ surfaces, as indicated by prevalent cell membrane damage. The bacteria on Ti_3_C_2_T_x_ film have a rough surface (Fig. 3B), indicating their damage, and some bacteria were even totally burst. Previously, we provided evidence that bacterial cells were ruptured by MXene nanosheets, which could cause the leakage of internal cell contents^5^. It was found that the number of bacterial cells in contact with Ti_3_C_2_T_x_/PVDF decreased as compared with that of PVDF control. As expected, the rough Ti_3_C_2_T_x_ film caused rupture of bacterial membrane and the intracellular densities of *E*. *coli* and *B*. *subtilis* decreased as seen from the higher magnification panels, revealing that they lost some intracellular substance^5^. This indicates that the MXene membrane can inhibit the bacterial growth and efficiently hinder the biofilm formation, which is important for water purification membranes.

**Figure 4:**
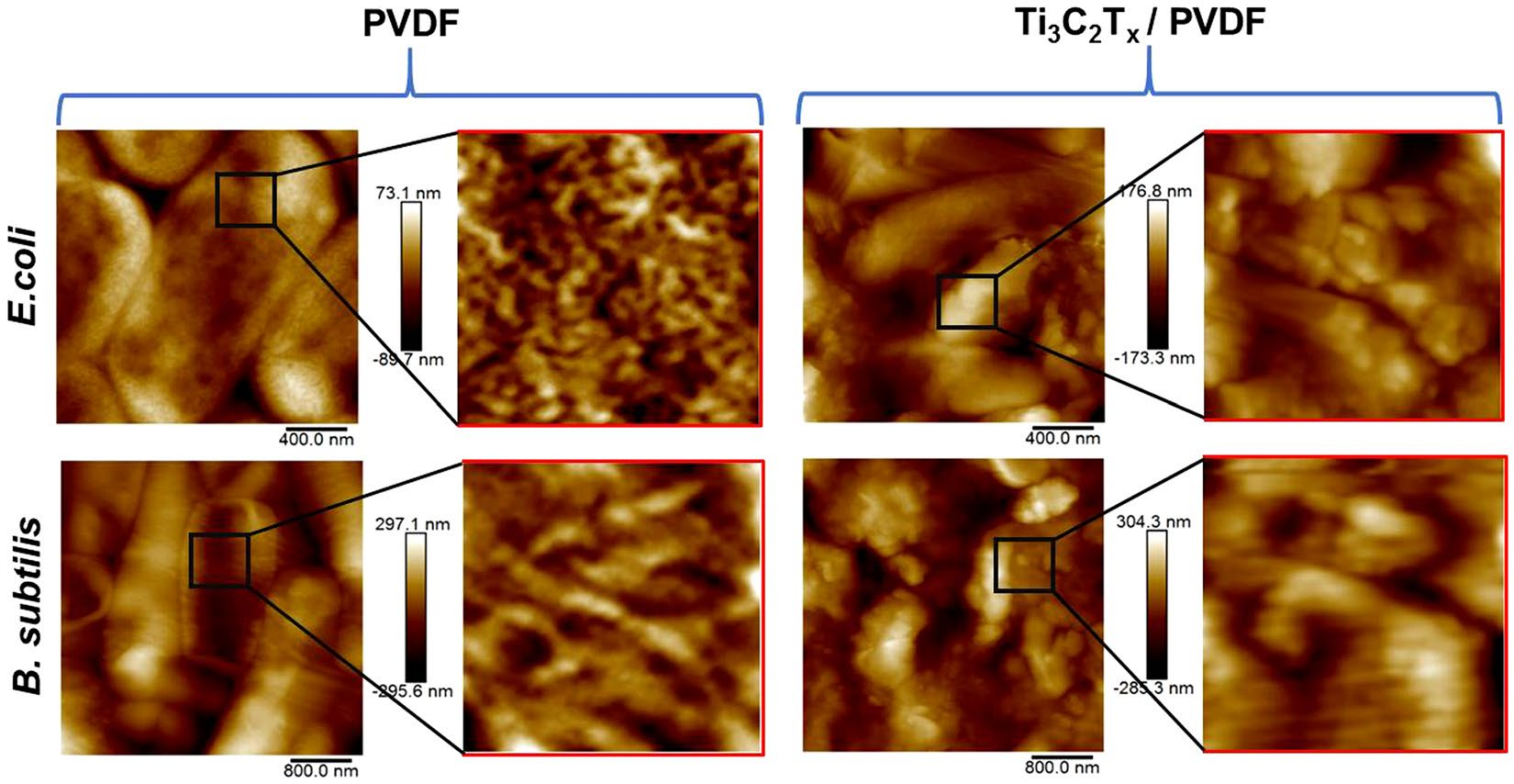
AFM images of *E*. *coli* and *B*. *subtilis* colonies grown on PVDF and Ti_3_C_2_T_x_ modified PVDF membranes. The black square areas indicate where a 0.5 × 0.5 μm zoom scan has been performed (right hand of each panel); showing fine detail of the bacterial capsule surfaces.

As further evidence, AFM scans were collected for the bacteria grown on the Ti_3_C_2_T_x_/PVDF as well as pristine PVDF membranes. Representative micrographs are shown in Fig. 4. For the control PVDF membranes, intact bacteria can be seen on the surface for both *E*. *coli* and *B*. *subtilis*. However, bacteria on Ti_3_C_2_T_x_ film were smaller and showed signs of structural damage. 0.5 μm images were cropped and split from bacterial surface scans and re-flattened, to allow evaluation of the bacterial surfaces, independent of the underlying membrane support. For *E*. *coli* on the control surface, the structure of the bacterial wall resembles the porous ‘filigree’ structure of peptidoglycan previously reported by other researchers^43^. For corresponding images obtained from *E*. *coli* on Ti_3_C_2_T_x_, this structure is not apparent, replaced by a less ordered globular arrangement of components, suggesting damage has occurred to the outer structure of the cell. For *B*. *subtilis* on the control surface a different (filamentous) surface structure is seen, compared to that observed for *E*. *coli*. It is similar to the ‘cabling’ structures formed from peptidoglycan previously observed by other researchers for *B*. *subtilis* ^44^. However, it must be noted that the cabling arrangements were previously observed on the *inner* surface of the cells alone. For images obtained from the bacterial surface on the Ti_3_C_2_T_x_ film, the surface structure appears to be less ordered.

Roughness values on the surface of bacterial membranes for both *E*. *coli* and *B*. *subtilis* are reported in Table 1 (mean values of 9 measurements for each sample). In both cases the root mean squared height (Sq) values increased for the bacteria on Ti_3_C_2_T_x_ compared with PVDF surfaces, indicating the change in bacterial surface morphology. Student’s t-tests were carried out, assuming two-tailed sample distributions with unequal variances, with the change to the *E*. *coli* surfaces being found to be more significant (*p* < 0.001) than for the *B*. *subtilis* (*p* = 0.06). The low significance of the Sq difference for *B*. *subtilis* does not reflect the different surface structure seen in the height images, where the surfaces appear very different. It should be noted that Sq is essentially the standard deviation of the heights of image data points^9^. As a result it is possible to have surfaces with different topographies having similar values of Sq.

**Table 1:** Roughness values on the surface of bacterial membrane for both *E*. *coli* and *B*. *subtilis*.

	RMS Roughness (nm)	SD	Deformation (nm)	SD
*E*. *coli*@PVDF	7.03	2.01	3.33	0.33
*E*. *coli* @Ti_3_C_2_T_x_/PVDF	22.69	9.18	6.08	2.63
*B*. *subtilis*@PVDF	12.55	3.52	3.88	0.55
*B*. *subtilis*@Ti_3_C_2_T_x_/PVDF	23.07	14.09	15.84	5.82

The PeakForce operating mode of the AFM allows access to nano-mechanical data simultaneously to height imaging. The mean values of the sample deformation for the same areas as for the Sq data are presented in Table 1. Each value represents an average of 9 image-mean values. The deformation values are the calculated penetration depth of the probe into the sample surface at the peak load values for each tip-sample interaction event, with the peak force kept at a constant set-point for all measurements (90 nN). As such, a higher deformation value represents a less rigid surface with a lower elastic modulus. For both bacteria, the deformation was significantly greater for bacteria on Ti_3_C_2_T_x_ surfaces than for those on the PVDF control. A two-sample t-test assuming unequal variances showed a high degree of statistical significance for both bacteria, with *p* values of 0.015 and 0.0003 for the *E*. *coli* and *B*. *subtilis* samples, respectively. This “softening” of the bacteria is most likely a result of the damage to bacterial cells observed in the SEM and AFM imaging and reflects the decrease in the cells viability due to interaction with the Ti_3_C_2_T_x_ film.

**Figure 5:**
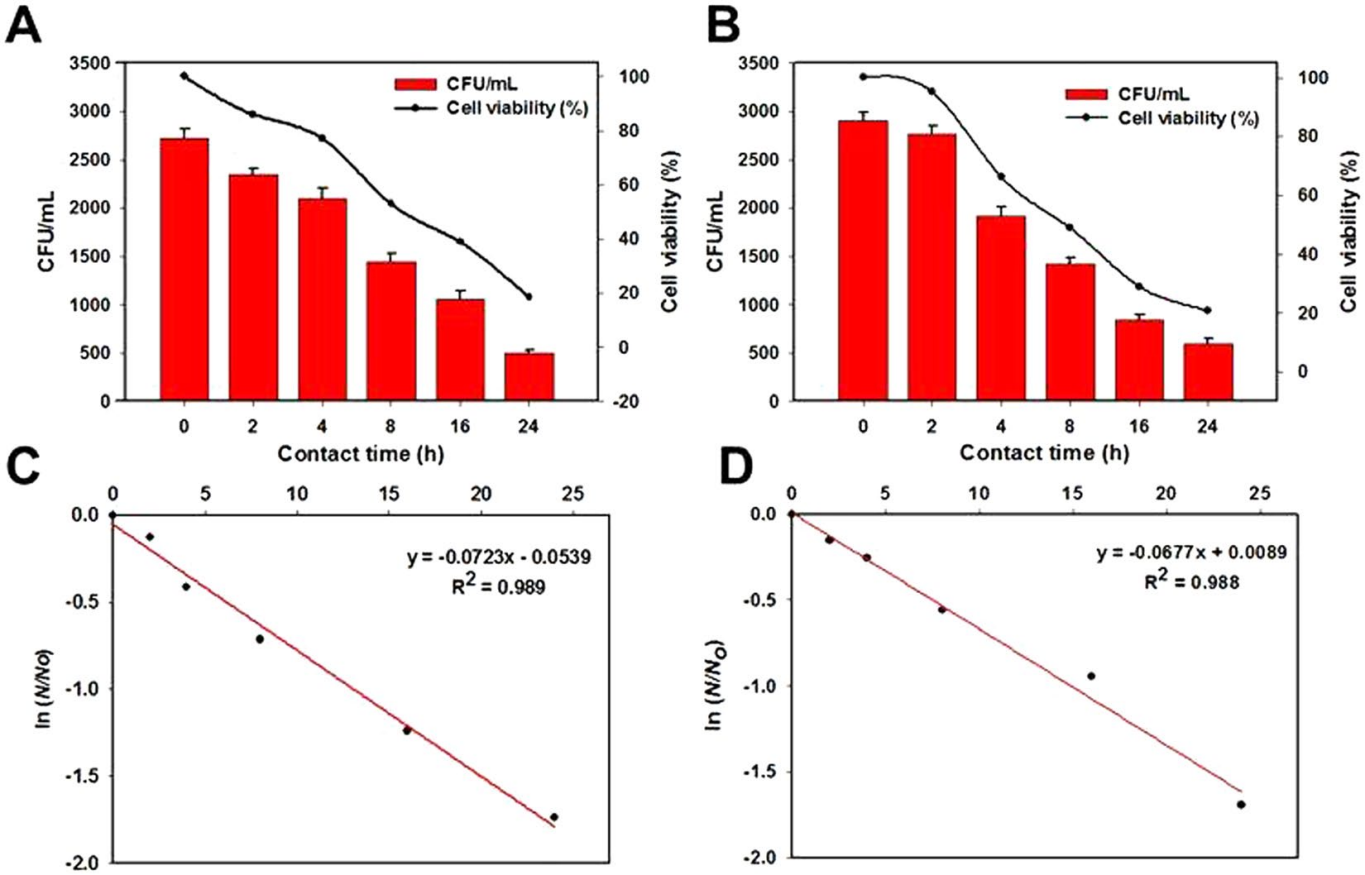
Cell viability measurements of (**A**) *E*. *coli* and (**B**) *B*. *subtilis* exposed to MXene membranes at different time intervals during 24 h of contact time. Survival rates were obtained by the colony forming count method as compared to that of control PVDF membrane. Error bars represent the standard deviation. First order rate plot for the inactivation of (**C**) *E*. *coli* and (**D**) *B*. *subtilis*.

Next, we evaluated the antimicrobial kinetics of Ti_3_C_2_T_x_ coated membranes. A series of batch shake experiments were performed with *E*. *coli* and *B*. *subtilis* suspensions exposed to Ti_3_C_2_T_x_/PVDF and PVDF membranes. Figure 5 depicts the growth of bacteria after exposure to Ti_3_C_2_T_x_ coated membrane at different time intervals which is measured based on the colonies growth on agar nutrient media. There was a lower bactericidal activity within the first two hours of incubation in both *E*. *coli* and *B*. *subtilis*. However, an evident decrease was observed in bacterial colonies growth on Ti_3_C_2_T_x_ films with the progression of time. After 24 h of contact time, the bacterial cell viability for both *E*. *coli* decreased to 18 ± 2.23%, whereas, for *B*. *subtilis* it was approximately 14.45% as compared to that of control PVDF membrane. This shows the pronounced inactivation of both bacterial strains. According to these results, Gram-negative bacteria seemed to have a higher resistance against MXene membranes as compared to Gram-positive bacteria. These findings are in line with the previous studies for Ti_3_C_2_T_x_ MXene dispersions^5^.

Based on the experimental data, the first-order Chick’s law^45^, which expresses the main principles of bacterial inactivation kinetics, was tested to determine the inactivation rate constants. Figure 5(C and D) depicts the bactericidal kinetics of MXene coated membranes as compared to that of PVDF control. The rate of inactivation of *E*. *coli* and *B*. *subtilis* was calculated using Chick’s law, with *ln*(*N*/*N*_*0*_) plotted as a function of time; where *N* is the number of bacterial cells at a given time and *N*_0_ is the initial bacterial cell count. The first order rate constant, *k*, was calculated per unit area of membrane and the inactivation rate for *B*. *subtilis* was 0.0723 h^−1^.cm^−1^ - somewhat higher compared to that of *E*. *coli* (0.0677 h^−1^.cm^−1^).

### Cytometric measurement of cell membrane damage

**Figure 6:**
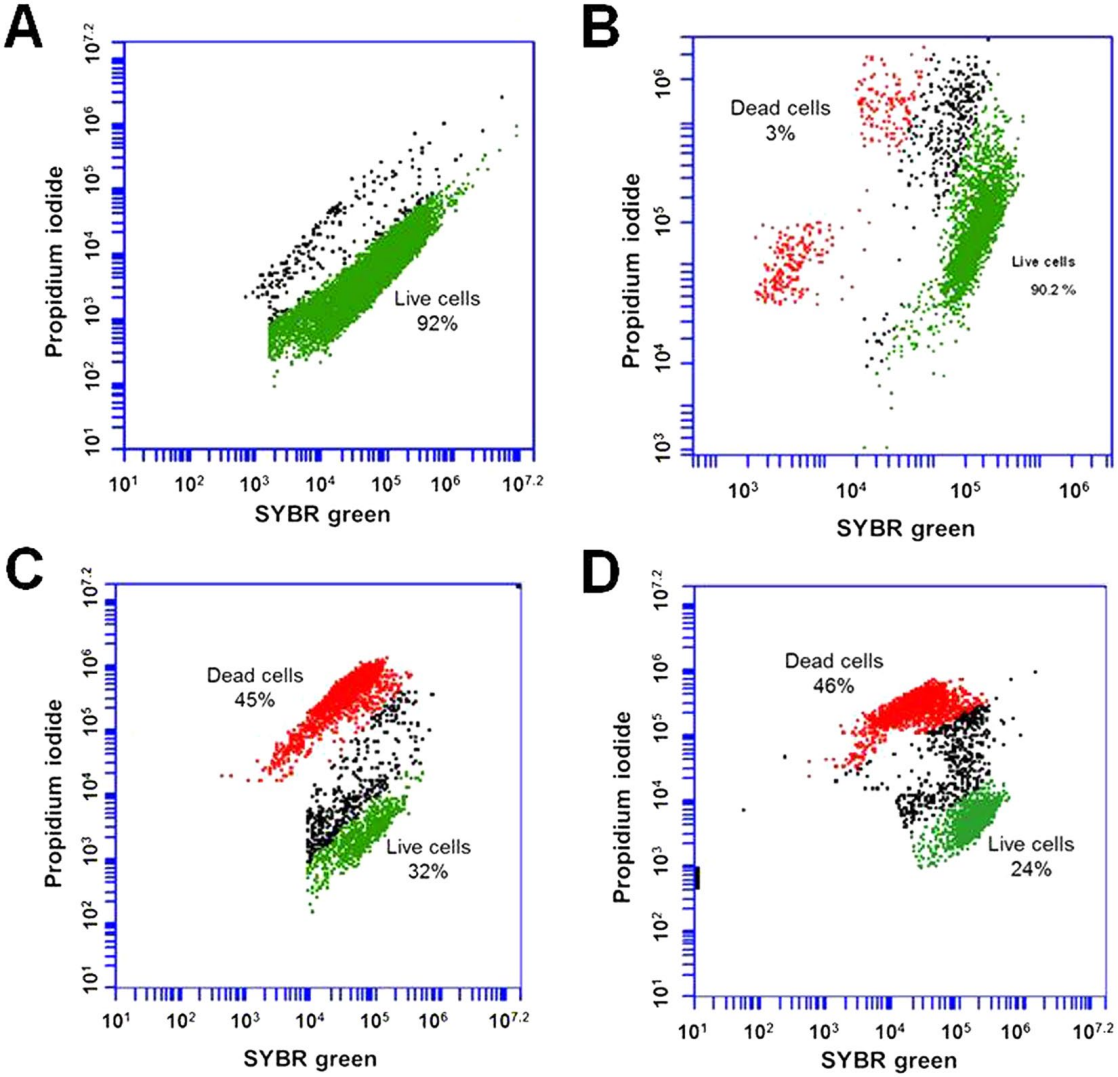
Cell viability measurement of *E*. *coli* and *B*. *subtilis* exposed to PVDF and Ti_3_C_2_T_x_. Flow cytometry dot plots of *E*. *coli* (**A**,**C**) and *B*. *subtilis* (**B**,**D**) bacterial cells exposed to control PVDF (**A** and **B**) and MXene films (**C**,**D**) for 24 h.

A mechanistic investigation of the interaction of Ti_3_C_2_T_x_ nanosheets with both bacterial cells was obtained from the flow cytometric analysis. The measurements of fluorescence reveal the populations of live, compromised and dead cells, leading to better understanding of the working of the antimicrobial agent. The dot plots in Fig. 6 show populations of *E*. *coli* and *B*. *subtilis* cells stained with PI and SYBR green, measured by flow cytometery after 24 h exposure to Ti_3_C_2_T_x_ coated membranes. Figure 6 shows the particular fluorescence patterns of *B*. *subtilis* and *E*. *coli*, doubly stained with SYBR green and PI. The low red and strong green fluorescence intensity region (P1) depicted the proportion of live bacteria, and the weak green and heavy red fluorescence intensity region (P2) indicated the proportion of the dead cells. Bacterial cells exposed to Ti_3_C_2_T_x_ coated membranes for 24 h showed a shift of population from viable to dead and compromised cells. For *E*. *coli*, 92.0% of cells with control PVDF membrane fell in the region P1, whereas the P1 values were 32% for bacteria exposed to Ti_3_C_2_T_x_ film. In the case of *B*. *subtilis*, the control presented 90% in P1 region (live cells), 3% in P2 region and remaining of compromised bacteria. Bacterial cells exposed to Ti_3_C_2_T_x_ film showed about 45% population of dead cells after 24 h of exposure for both *E*. *coli* and *B*. *subtilis*, whereas, almost 30% of the total cell population of both bacterial strains seemed to have been compromised. The group of bacteria in contact with PVDF control exhibited a much smaller number of compromised cells. Results showed negligible amount of dead or lysed cells of *E*. *coli* and *B*. *subtilis* in the control samples. Bacterial permeability to propidium iodide (PI) indicated the alteration and occurrence of substantial damage to the cell membrane, which finally causes cell death^46^. Ti_3_C_2_T_x_ coated membrane caused irreparable damage to the bacterial cell membrane resulting in growth inhibition.

Evidenced by flow cytometry, SEM and AFM analysis, a thin layer of Ti_3_C_2_T_x_ MXene sheets on the membrane surface seems to be very efficient in inhibiting bacterial growth. Surface oxidation of aged Ti_3_C_2_T_x_ coated membranes demonstrated higher antibacterial efficiency as compared with the fresh membrane. This is most likely attributed to formation of anatase TiO_2_ nanocrystals and highly defective 2D carbon structure^47^, which helps in bacterial inhibition, possibly by the direct physical contact of MXene and TiO_2_ sharp edges (see ref. 47) with the bacterial surface, thereby causing physical stress and disruption of cellular membranes. Also, oxidative stress cannot be ruled out in case of aged Ti_3_C_2_T_x_ membranes since TiO_2_ can stimulate the oxidative stress on bacterial surface due to possible radicals formation^37^. A number of studies have explored the antibacterial activity of membranes based on nanomaterials like carbon nanotubes (CNTs), graphene and their nanocomposites used for filtration and separation applications^11, 48^. Similarly, graphene oxide coatings can significantly improve the antibacterial properties of commercial membrane filters^49^. The primary antibacterial activity of the nanomaterial modified membranes in the studies discussed above have been explained by the direct contact of nanosheets and/or nanotubes with the bacterial surface, thus causing physical stress and membrane damage. The hydrophilicity of the Ti_3_C_2_T_x_ surface may facilitate inactivation of bacteria by direct contact interaction. The high density of defects on MXene could also enhance the antibacterial activity^48, 50^. Also, MXene surfaces are covered by functional groups, some of which can be quite reactive, especially in contact with basic environment (e.g., Ti-F)^41^. Ti_3_C_2_T_x_ MXene has also been shown to adsorb and degrade positively charged dye molecules^25^. It’s a strong reducing agent and a variety of chemical interactions with bacterial membranes may be possible, but they need further studies. Here we introduce the potential of MXene as a material for anti-biofouling membranes; however, a comprehensive understanding of MXene’s behavior under different operating environments is important for future applications in water/wastewater treatment. For instance, controlling the functional groups, lateral size, number of layers, and surface and edge properties of MXene nanosheets may allow controlling its antibacterial activity. Ti_3_C_2_T_x_ MXene is only one of about 20 MXenes reported to date and new structures are added to the list every couple of months^21^.

## Conclusions

Hydrophilic Ti_3_C_2_T_x_ MXene coatings have excellent antibacterial activity against both, *E*. *coli* and *B*. *subtilis*. Surface oxidation of aged membrane showed a significant improvement of antibacterial activity as compared with the freshly prepared membranes. This has been attributed to the synergistic effect between Ti_3_C_2_T_x_ nanosheets and TiO_2_/C formed on the surface. As evidenced by flow cytometery, colony forming counts, SEM and AFM analysis, MXene inactivated the bacterial growth and caused cell death on the membrane surface. MXenes, as a new family of 2D materials, may open a door for developing efficient antibacterial membranes for water and wastewater treatment, as well as other applications.

## Materials and Methods

### Synthesis, delamination and dispersion of Ti_3_C_2_T_x_ MXene

A colloidal solution of single- and few-layer Ti_3_C_2_T_x_ particles was obtained by delaminating the multilayer Ti_3_C_2_T_x_ powders by ultrasonication after etching Ti_3_AlC_2_ MAX phase with LiF/HCl solution, as described previously, with minor modifications in the process^32^. Briefly, the obtained Ti_3_C_2_T_x_ powder was dispersed in deaerated water with a weight ratio of Ti_3_C_2_T_x_:water of 1:250. The suspension was sonicated under flowing argon and then centrifuged for 1 h at 3000 rpm to obtain the supernatant containing Ti_3_C_2_T_x_ flakes. TEM, SEM, energy-dispersive X-ray spectroscopy (EDX), and XRD were used to confirm the morphology of the flakes.

### Preparation of Ti_3_C_2_T_x_ membranes

The MXene membranes were prepared by the VAF. Typically, the delaminated Ti_3_C_2_T_x_ solution was diluted to 0.01 mg.mL^−1^ and filtered through a commercial PVDF membrane (Hydrophilic, 0.45 µm pore size, EMD Millipore Durapore, US) with a diameter of 47 mm. The membranes with different thickness were prepared by depositing 2, 4 and 6 mg of Ti_3_C_2_T_x_ on 47 mm diameter PVDF to obtain average thickness of 0.6, 1.2, and 1.8 µm, respectively. A glass microfiltration apparatus, with a fritted alumina supported base 40 mm diameter, was used for VAF. The filtered membranes were air-dried and used for further antibacterial experiments on the PVDF support.

### Cell cultures

The antibacterial properties of Ti_3_C_2_T_x_ membranes were evaluated using *E*. *coli* and *B*. *subtilis* as the model gram-negative and Gram-positive bacteria, respectively. Glycerol stocks were used to inoculate defined overnight cultures in Luria-Bertani broth (LB) medium at 35 °C. Following that, 1 mL of cell suspensions were sub-cultured and harvested during the exponential growth phase. Cultures were centrifuged at 5000 rpm for 5 min and pellets obtained were washed three times with phosphate buffer saline (PBS) (pH = 7.2) to remove residual macromolecules and other growth medium constituents. The cell pellets collected by centrifugation were re-suspended in sterilized PBS and diluted to cell concentration of approximately 10^7^ colony forming units (CFU).mL^−1^.

### Evaluation of antibacterial activity

The antibacterial activity of Ti_3_C_2_T_x_ modified membranes was investigated against Gram-negative bacteria *E*. *coli* and Gram-positive bacteria *B*. *subtilis* by two methods: bacterial filtration through membranes and direct bacterial suspensions with MXene films. In first assay, both *E*. *coli* and *B*. *subtilis* cells were diluted to 10^4^ CFU.mL^−1^ in PBS. Typically, 50 µL of bacterial suspensions were further diluted in PBS to make total 10 mL solution and after this suction-filtered through the PVDF and MXene modified membranes. Membranes with bacteria on the surface were air dried for 10 min and placed on LB agar plates and incubated overnight at 35 °C. Finally, the growths of the colonies on the membrane surface were observed. The filtrate was also collected for subsequent analysis of bacterial cell presence by spreading 100 µL on agar nutrient media. The filtration assembly and glassware used were autoclaved and the membrane samples were sterilized with UV irradiation for 30 min before the experiments. To avoid the contamination of *E*. *coli* and *B*. *subtilis* cells, separate filtration assemblies were used for each bacterial assay. To avoid rupture of the membranes, the filtration was operated under a low pressure of about 2 kPa. After incubation for 24 h at 35 °C, the number of colonies grown on membrane surface was counted to determine the antimicrobial efficiency of Ti_3_C_2_T_x_ membranes. To investigate the effect of Ti_3_C_2_T_x_ membrane aging and surface oxidation on its antibacterial activity, the Ti_3_C_2_T_x_ modified membranes were prepared and allowed to age by exposing to ambient atmosphere for over a month. The antibacterial characteristics of aged membranes were studied as described above.

In the second set of bactericidal assay, several samples of Ti_3_C_2_T_x_ coated membranes (1.5 cm^2^) were suspended in *E*. *coli* and *B*. *subtilis* (10^7^ CFU.mL^−1^) in saline and incubated at 35 °C under constant shaking at 110 rpm for up to 24 h. A control assay with bacteria without any membrane was used as a negative control, and the second with the bacteria in saline in the presence of PVDF membrane was used as a positive control. To examine the effect of contact time on bactericidal activity, membranes were taken out at 2, 4, 8, 16 or 24 h of incubation and gently washed with saline solution to remove loosely bound bacteria. The membranes were then placed in 2 mL of PBS and sonicated for 2–3 min to detach the bacteria from the membrane surface. The viability of cells attached to the membrane surface was analyzed by spreading 100 μL of suspension on nutritive agar plates after overnight incubation at 35 °C. The experiments were carried out in triplicate and average values were reported. The antibacterial activity was calculated using the following equation:

$$R=\frac{({N}_{c}-{N}_{m})}{{N}_{c}}\times 100,$$

where, *N*_c_ and *N*_m_ correspond to the number of colonies incubated with the PVDF (control) and MXene modified membranes for a given duration of treatment, respectively.

### Flow cytometric assay for bacterial viability analysis

Viability of bacterial cells and disruption of membrane integrity were evaluated using propidium iodide (PI), and SYBR green. PI enters only permeable cells, binds DNA, and fluoresces at 620 nm, when stimulated by a laser at 488 nm whereas SYBR green can stain the total bacteria. Briefly, several tubes containing 2 mL (1 × 10^7^ CFU.mL^−1^) of the *E*. *coli* and *B*. *subtilis* cells and membranes (1.5 cm^2^) were incubated at 35 °C for 24 h. After incubation, the membranes were gently washed with PBS to remove loosely bound bacteria. Washed membranes were placed in 2 mL of PBS and sonicated for 2–3 min to detach the bacteria from the membrane surface. Viability of the cells attached to the membrane surface was analyzed by flow cytometery. SYBR green (10,000 × stock) (10 µL) was mixed with 30 µL PI (20 mM) into 1.0 mL of sterile dH_2_O and vortexed thoroughly. All the samples were stained with PI and SYBR green to achieve the concentration of 0.3 mM and 1x, respectively. Samples were analyzed by a flow cytometer (BD CSampler, Accuri,). They were illuminated with a 15 mW argon ion laser (488 nm), and the fluorescence was detected via 525 ± 10 nm (green) and 620 ± 10 nm (red) band pass filters. Signals were amplified with the logarithmic mode for side scattering, forward scattering, and fluorescence. In dot plots of fluorescence, different bacterial populations were gated according to the viability stages.

### Cell morphology observation with SEM and AFM

SEM analysis was performed to observe the effect of Ti_3_C_2_T_x_ MXene on morphology and surface structure of the bacterial cells using FEI-Nova Nano SEM 650. SEM imaging of samples was accomplished using the following procedures. Following the experiments, cells on both PVDF and Ti_3_C_2_T_x_ coated membrane surfaces were fixed with 2.5% glutaraldehyde for 4 h at 4 °C, followed by washing with 0.1 M phosphate buffer (pH 7.4) and dehydration with a graded ethanol series (25, 50, 80, 100%). Samples were allowed to dry completely at room temperature and were then coated with gold by sputtering (5 nm).

AFM characterization was carried out using a Dimension Icon model AFM with NanoScope V Controller (Bruker AXS, USA) operating in PeakForce mode. All measurements were made in ambient conditions using NSG30 silicon tapping mode probes (NT-MDT, Russia). Samples, which had been fixed using the identical procedure for that of SEM samples but without gold sputter-coating step, were immobilised onto glass sides using double-sided tape, which was in turn fixed onto the sample stage using instrument vacuum. All height images were flattened using 2^nd^ order levelling to remove background offset and sample tilt prior to further analysis.

## Electronic supplementary material

Supplemental
