# Supplementary material for: Efficient Antibacterial Membrane based on Two-Dimensional Ti3C2Tx (MXene) Nanosheets
Source: Sci Rep. 2017 May 9;7:1598. doi: 10.1038/s41598-017-01714-3 (PMC5431673; doi:10.1038/s41598-017-01714-3)
Supplement: Supplementary file 1 — Supplemental [file 41598_2017_1714_MOESM1_ESM.pdf]

Supplemental Material

Efficient Antibacterial Membrane based on Two-Dimensional $Ti_3C_2T_x$ (MXene) Nanosheets

Kashif Rasool[1], *Khaled A. Mahmoud*[1]*, *Daniel J. Johnson*[1], *Mohamed Helal*[1], *Golibjon R. Berdiyorov*[1], *and Yury Gogotsi*[2]

[1]Qatar Environment and Energy Research Institute (QEERI), Hamad Bin Khalifa University (HBKU), P.O. Box 5825, Doha, Qatar. Fax: +974 44541528

[2]Department of Materials Science and Engineering and A.J. Drexel Nanomaterials Institute, Drexel University, Philadelphia, PA 19104, USA

E-mail: kmahmoud@hbku.edu.qa

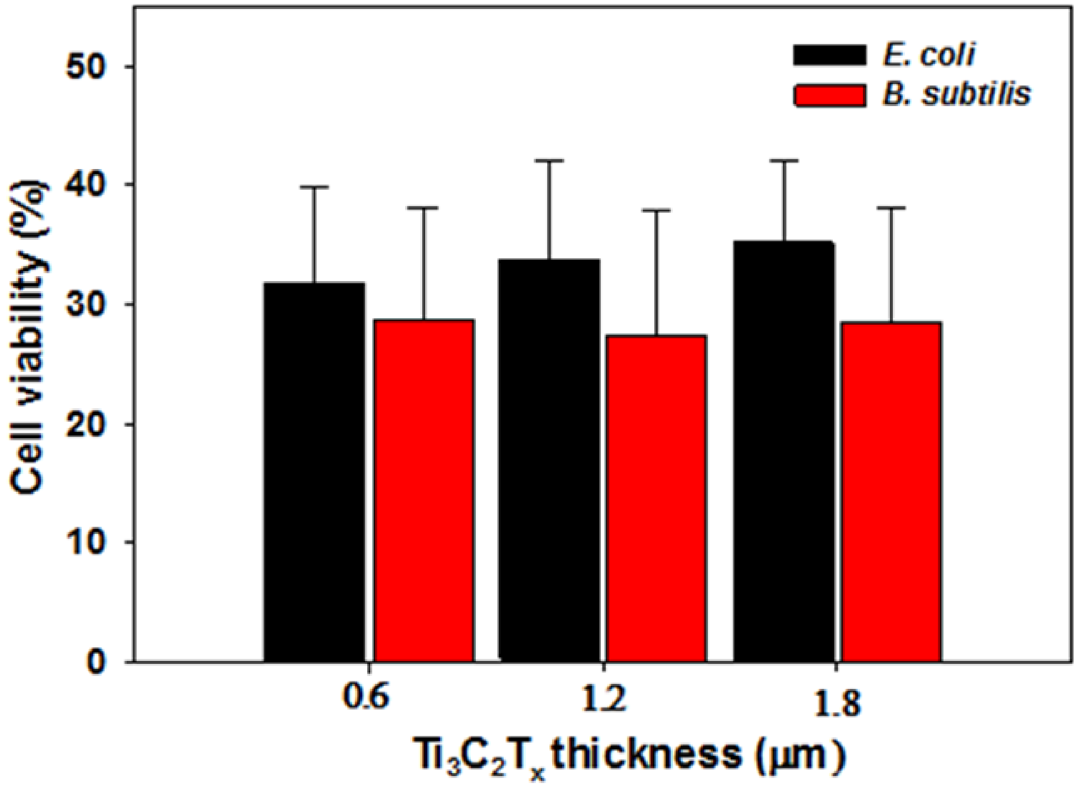

Figure S1: Antibacterial activity of freshly prepared $Ti_3C_2T_x$ membranes. Cell viability measurements of *E. coli* and *B. subtilis* grown on different thickness of $Ti_3C_2T_x$ MXene coated PVDF membranes. Survival rates were obtained by the colony forming count method.
